# POCUS, how can we include the brain? An overview

**DOI:** 10.1186/s44158-022-00082-3

**Published:** 2022-12-27

**Authors:** Juliana Caldas, Carla Bittencourt Rynkowski, Chiara Robba

**Affiliations:** 1 Escola Bahiana de Medicina e Saúde Pública, Salvador, Brazil; 2 Instituto D’Or de Pesquisa e Ensino (IDOR), Salvador, Brazil; 3 Salvador, Brazil; 4 Intensive Care Unit of Cristo Redentor Hospital, Porto Alegre, Brazil; 5 Intensive Care Unit, Hospital Ernesto Dornelles, Porto Alegre, Brazil; 6 Italy Anesthesia and Intensive Care, Policlinico San Martino, IRCCS for Oncology and Neuroscience, Genoa, Italy; 7 Dipartimento di Scienze Chirurgiche Diagnostiche Integrate, University of Genoa, Genoa, Italy

**Keywords:** POCUS-BU, Brain ultrasound, Cerebral ultrasound, TCD, TCCD, Neurosonology

## Abstract

Point-of-care ultrasound (POCUS) is an essential tool to assess and manage different pathologies in the intensive care unit, and many protocols have been proposed for its application in critical care literature. However, the brain has been overlooked in these protocols.

Brain ultrasonography (BU) is easily available, and it allows a goal-directed approach thanks to its repeatability and immediate interpretation and provides a quick management and real time assessment of patients’ conditions. Based on recent studies, the increasing interest from intensivists, and the undeniable benefits of ultrasound, the main goal of this overview is to describe the main evidence and progresses in the incorporation of BU into the POCUS approach in the daily practice, and thus becoming POCUS-BU. This integration would allow a noninvasive global assessment to entail an integrated analysis of the critical care patients.

**Supplementary Information:**

The online version contains supplementary material available at 10.1186/s44158-022-00082-3.

## Introduction

Point-of-care ultrasound (POCUS) is an essential tool in intensive care units to assess and manage pathologies of the heart, lungs, vessels, and abdomen [1–3]. Several protocols, such as Rush, Fast, and LUS, have been included in the critical care practice [1–3]. Nevertheless, the brain has been overlooked in these protocols.

A recent consensus of experts’ recommendations regarding the basic skills for “head to toes” ultrasonography in the intensive care setting included in its recommendations also the use of the brain ultrasound (BU) for triage or clinical suspicion for intracranial hypertension [4]. Although there is a lack of conclusive evidence surrounding BU and therefore the panel was unable to provide strong recommendations on the majority of circumstances related to the brain [4], this consensus paved the way for the inclusion of BU in ultrasonography evaluation of critically ill patients. Over the last decade, there has been a rising interest in this field and updated studies have been consistently published showing that BU can be an important technique to routinely provide imaging and assess cerebrovascular alterations [5–8].

BU is considered a relatively simple method and short-term learning program, where reliable results can be achieved even if conducted by inexperienced operators [9, 10]. Nevertheless, BU is characterized by some limitations, such as the necessity of patent transcranial acoustic windows and operator dependency. To address this concern, a recent consensus aimed to define a standardized approach for the use of BU, defining the various levels of skills/competencies associated with this technique [11].

Besides the well-known benefits of being non-invasive, safe, and bedside [12], the advantage of bringing physicians and intensivists closer to the patient is unquestionable, as it forces them to constantly reassess the efficiency of their management. Incorporating the BU to POCUS (POCUS-BU) could add another piece of the puzzle to complement the global ultrasound assessment.

Therefore, the main aim of this review is to describe the techniques and applications for POCUS-BU in critical care patients, as well as provide evidence of its utility in guiding clinical management in intensive care units.

## How it is performed

There are two existing types of BU evaluation: traditional transcranial Doppler (TCD), which provides information regarding the cerebral blood flow velocity of the main vessels, and transcranial color-coded duplex Doppler sonography (TCCD), which combines B-mode and color Doppler imaging.

### TCD ultrasonography

TCD was first described in 1982 by Aaslid et al. [13] as a readily available, noninvasive, and reproducible technique used to evaluate cerebral blood flow (CBFV) hemodynamics through the insonation of the basal cerebral arteries, usually performed with a 2–2.5 MHz probe.

**Fig. 1:**
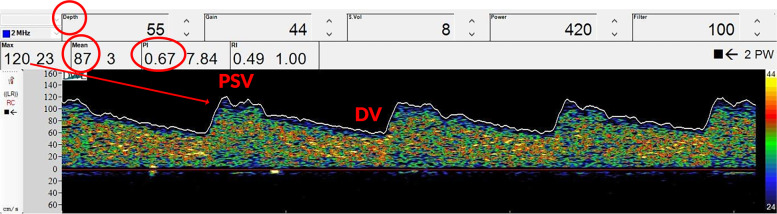
Representation of the transcranial Doppler ultrasonography showing cerebral blood flow velocity. PSV, peak systolic velocity; DV, diastolic velocity; MFV, mean flow velocity

The TCD technique is based on the *Doppler* effect, according to which a sound wave, emitted with a certain frequency, strikes a moving object (e.g., red blood cells moving in an insonated vessel), and produces a reflected wave with a different frequency (the *Doppler* shift), directly proportional to the velocity of the object [10]. The normal spectral waveform has a characteristic shape: a sharp systolic upstroke and stepwise deceleration with positive end-diastolic flow (Fig. 1) resulting in a peak systolic velocity (PSV in cm/s), end-diastolic velocity (EDV in cm/s), and mean flow velocity (MFV in cm/s). The POCUS-BU can therefore analyze the waveform itself, as certain pathologies can alter FV waveform, but it cannot provide any information on cerebral anatomy.

### Windows for insonation

#### Transtemporal approach

This view is obtained by positioning the probe between the tragus and the lateral orbit wall, with the probe marker facing toward the eye. The first step of POCUS-BU using TCD could be to assess the CBFV of the middle cerebral artery (MCA) through the transtemporal window, which has been considered by a recent expert consensus as a basic skill for BU [11]. MCA is responsible for approximately 70% of the flow from internal carotid artery (ICA) and 70 to 80% of CBF comes from bilateral ICA. Therefore, the analysis of MCA bilaterally can bring enough information about CBF. The additional acoustic windows are also accessible through BU; however, this requires a more advanced training and is considered a basic-plus skill [11].

**Fig. 2:**
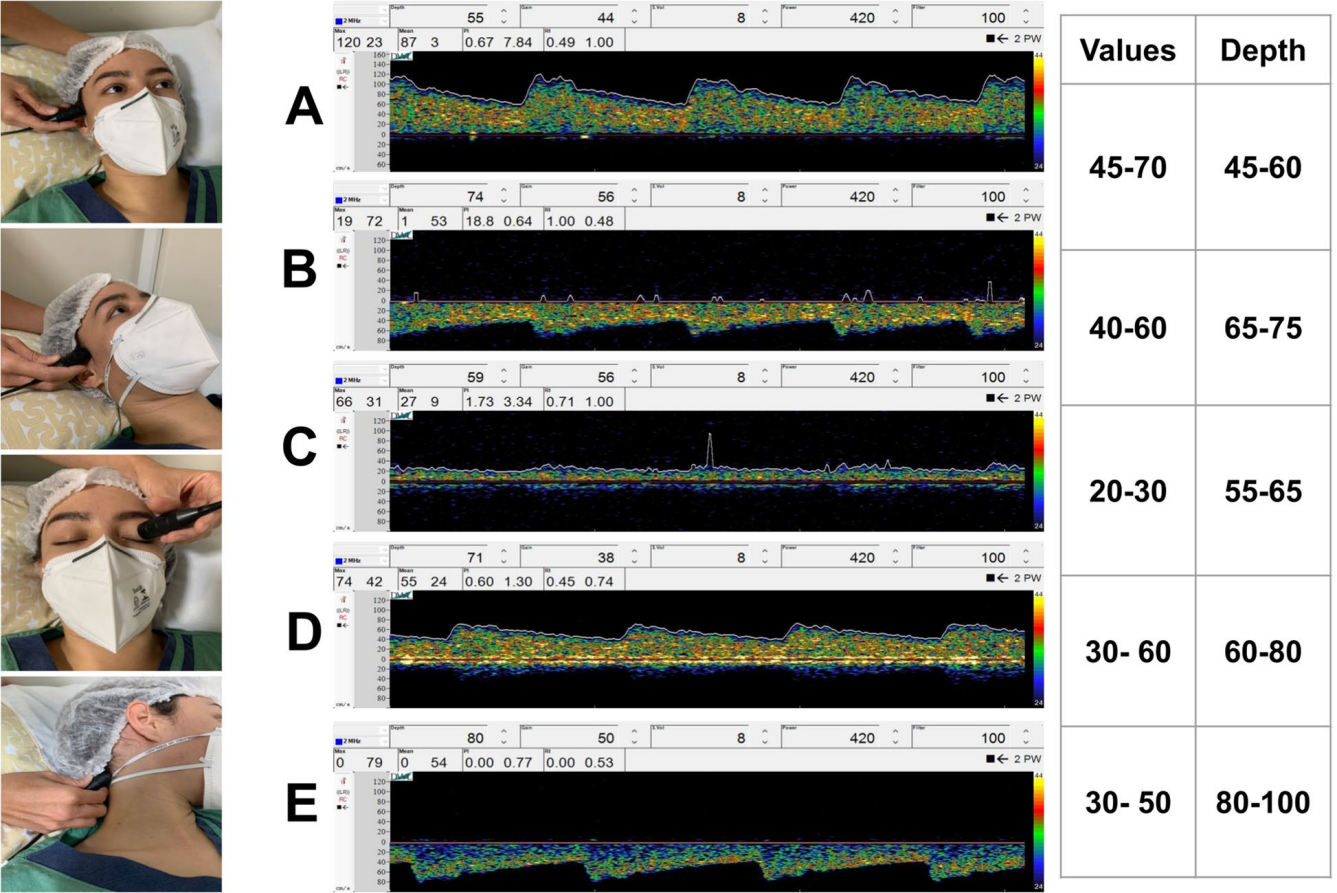
The figure represents the transtemporal, transorbital, and suboccipital bone window and the normal curve of blood flow velocity with its pattern, depth, value, pulsatility, and resistance index. **A** Transtemporal window and middle cerebral artery in 55mm of depth. **B** Anterior cerebral artery in 74mm of depth. **C** Posterior cerebral artery in 59mm of depth. **D** Transorbital window and siphon artery in 70mm of depth. **E** Suboccipital window and Basilar artery in 80–110mm of depth

It is important to note that POCUS-BU emphasizes measurements of the MCA, although in this window it is possible to evaluate the anterior cerebral artery (ACA), posterior cerebral artery (PCA), and anterior communicating artery (PCOM) as well. The depth, sharpness, and sound help to differentiate the curves of blood flow velocities (Fig. 2). Insonation of these arteries is considered a basic-plus skill [11].

#### Transorbital window

Transorbital insonation can be used to evaluate the ipsilateral ophthalmic artery and the ICA siphon. The transducer is placed gently over the eyelid and angled slightly toward the medial and upward (Fig. 2 (D)). The ICA siphon is also considered a basic-plus skill [11].

#### Suboccipital window

To obtain this view, the operator should turn the patient’s face to one side and place the transducer just below and medial to the mastoid process, directing the transducer slightly medially toward the bridge of the nose or contralateral eye.

The suboccipital window is key to obtain flow signals from the vertebral arteries (VA) and the basilar artery (BA). The latter may be visualized by aiming the transducer slightly upward and medially and increasing the depth. If the BA cannot be visualized, one may place the transducer just below the occipital protuberance and guide it toward the nasal bridge, commonly called the transforaminal window. The flow from the basilar artery is directed away from the transducer, producing a negative wave (Fig. 2 (B)). Insonation of the vessels in the cerebral posterior circulation is considered a basic-plus skill [11].

#### Submandibular window

Transducer should be placed laterally, under the jaw anterior and medial to the sternocleidomastoid muscle, directing the transducer upwards and slightly medially with a depth of 50mm. The distal ICA should be visualized as a low-resistance flow signal directed away from the transducer.

### TCCD ultrasonography

TCCD combines B-mode and color Doppler imaging. This technique provides some advantages compared to traditional TCD, due to its capability of assessing both the intracerebral vascular system and anatomical structures, either bone or parenchymal [12]. TCCD is usually performed using a 2–2.5-MHz sectorial transducer probe that allows visualization of the main cerebral structures and vessels. According to recent consensus, the insonation of transtemporal planes below is considered a basic-plus skill [11].

#### The mesencephalic plane

**Fig. 3:**
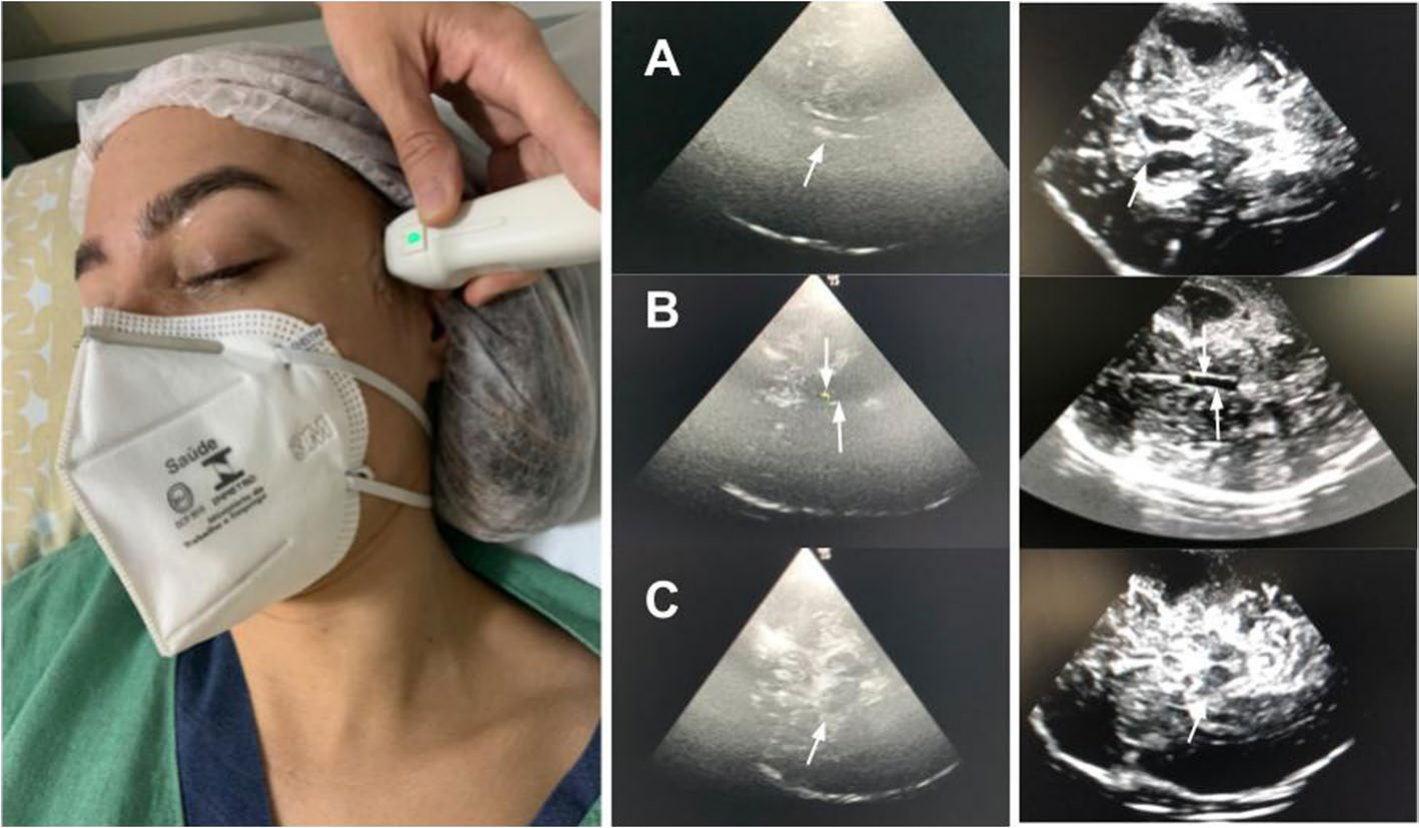
The transtemporal window approach and the three images of the brain’s ultrasonography planes. **A** Ventricular plan. **B** Diencephalic plan. **C** Mesencephalic plan

It is the most basal plane and allows the identification of the contralateral skull as principal landmark (usually at 12–15cm) confirming the presence of an adequate insonation window. The midbrain is usually easily identified at the midline, resembling a butterfly with the wings directed anteriorly (also called *Mickey face*) (Fig. 3 (C)).

#### Diencephalic plane

It can be evaluated from the mesencephalic plane. An approximate 10° cranial tilting of ultrasound beam allows the identification of the third ventricle, visualized as two pulsating parallel lines (usually less than 10mm apart) positioned slightly more cranially and anteriorly to the midbrain (Fig. 3 (B)).

#### Ventricular plane

In this plane, an additional cranial tilting of the ultrasound beam allows visualization of the thalamus and frontal horns of the lateral ventricles (Fig. 3 (A)).

The evaluation of these anatomical structures has been considered a basic-plus skill in a recent expert consensus on skill recommendations and competency levels in performing BU within the critical care setting [11].

### Main indexes obtained with TCD/TCCD

#### Pulsatility index (PI)

The resistance of intra-arterial flow can be assessed by measuring the PI, which is calculated by subtracting end-diastolic velocity from peak systolic velocity and dividing the resulting value by the mean flow velocity [14]. The PI is independent of the angle of insonation, and a value of more than 1.3 represents high resistance blood flow [14]. Furthermore, in some specific cases, this can be linked to high ICP (see the “Clinical applications” section and Fig. 5).

$$\textrm{PI}=\left(\textrm{PSV}-\textrm{EDV}\right)/\textrm{MFV}$$

#### Intracranial pressure estimation (ICPtcd)

Cerebral perfusion pressure (CPPe) and ICP estimation through TCD (ICPtcd) have been studied for more than 20 years [15]. Recent studies suggest that ICPtcd has high negative predictive value in ruling out intracranial hypertension and may be useful to clinicians in situations where invasive methods cannot be used or are unavailable [7, 16].

The intracranial pressure estimation by TCD (ICPtcd) formula is a mathematical model which comprises parameters derived from BU flow velocities and arterial blood pressure [15].

$${\displaystyle \begin{array}{c}\textrm{CPPe}=\textrm{MAP}\ast \textrm{CBFVd}/\textrm{CBFVm}+14\\ {}\textrm{ICPtcd}=\textrm{MAP}-\textrm{CPPe}\end{array}}$$

#### Lindegaard/Soustiel indexes

The BU could help in the diagnosis of cerebral vasospasm consequent to subarachnoid hemorrhage. The Lindegaard ratio or Lindegaard index (LI) has been proposed to differentiate between cerebral vasospasm and hyperemic flow [17]. The Lindegaard ratio is calculated using the CBFV of the MCA and the internal carotid artery (ICA). Furthermore, LI allows to graduate the vasospasm (Table 1).

**Table 1:** The vasospasm grade is related to the two indexes, Lindegaard and Soustiel

	Vasospasm grade
**MCA or ACA/ICA**	
< 3	Hyperemia
3–4	Mild
4–5	Moderate
5–6	Severe
**BA/ICA**	
2.5–3.0	Moderate
> 3.0	Severe

*MCA*, middle cerebral artery; *ACA*, anterior cerebral artery; *ICA*, internal carotid artery

The identification of vasospasm is considered a basic-plus skill [11].

$$\textrm{LI}=\textrm{MCA}\ \textrm{or}\ \textrm{ACA}/\textrm{ICA}$$

The Lindegaard index focuses on the anterior circulation vessels and does not address issues in the vertebrobasilar system. Basilar artery (BA) vasospasm has been defined as moderate when the CBFV was higher than 60cm/s and severe above 85cm/s. Similarly, in an attempt to differentiate between basilar hyperemia and vasospasm, the Soustiel index (SI) is calculated and analyzed with the ratio between BA and ICA (Table 1) [18].

$$\textrm{SI}=\textrm{BA}/\textrm{ICA}$$

##### Optic nerve sheath diameter (ONSD)

There is a strong anatomical relationship between the optic nerve and the subarachnoid space within the brain. The meninges surround the optic nerve, and consequently, a rise in ICP results in increased ONSD and optic disc height [19].

Ultrasound measurement of ONSD has been shown to correlate well with invasive measurements of ICP [19]. The majority of studies that measured ONSD as a means of diagnosing raised ICP were performed in patients with traumatic brain injury [20]. Nevertheless, this method has also been found to be useful in patients with various pathologies (Table 2).

**Table 2:** Summary of the major studies published in the last 5 years about ultrasonographic optic nerve sheath diameter

Author and year	Type of study	***N***	Type of patients	Cutoff (mm)	Main results and conclusion
Subramanian S. et al., 2021 [21]	Prospective observational	51	Patients with hydrocephalus and pre-procedure	5.5	ONSD on postoperative day 7 after CSF diversion correlates well with early surgical outcome. Rise in postoperative day 7 ONSD at follow-up correlates with failure of the CSF diversion procedure
Grupt and Pachisia, 2019 [22]	Prospective observational	100	All patients in whom lumbar puncture was indicated	6.3	There is a positive correlation of ONSD and CSF pressure. ONSD of >0.63 cm suggests a CSF pressure of >20 cm of water.
Agrawal, D et al., 2021 [23]	Prospective, blinded study of diagnostic accuracy	120	Consecutive patients with severe TBI	7.2	The optimal threshold was >0.72cm, with sensitivity 82% and specificity 79%. Optimal ODE threshold was >0.04cm, with sensitivity 90% and specificity 71%.
Çelik K et al., 2021 [24]	Prospective observational	162	TBI	-	A negative correlation was detected between ONSD values and GCS values and ONSD significantly increased in patients who died.
Xu H, et al., 2022 [25]	Prospective observational	529	Hemorrhagic stroke	5.9	ONSD of the poor outcome group was significantly greater than that of the favorable outcome group. ONSD improved the accuracy of ultraearly hematoma growth in the prediction of poor outcome by ROC curve.
Manouchehrifar M. et al., 2018 [26]	Prospective observational	80	Hemorrhagic and ischemic stroke	6.0	ONDS has moderate accuracy in differentiation of hemorrhagic and ischemic stroke
Yüzbaşıoğlu Y., et al., 2018 [27]	Prospective, cross-sectional	108	Cerebrovascular disorders	5.7	A positive relationship was determined between NIHSS scores and ONSD values. The specificity and sensitivity values were determined as 98.1% and 81.8%, respectively, for a cutoff value of 5 mm and as 100% and 72.7%, respectively, for a cutoff value of 6 mm
Wang Li-juan, et al., 2018 [28]	Prospective observational	60	Suspected of having elevated ICP for various reasons	5.8	ICP and ONSD values obtained on admission were strongly correlated. The dilated ONSDs decreased along with the elevated ICP reduction at follow-up 1 month.
Jeon J. P. et al., 2017 [29]	Prospective observational	63	Patients who required an external ventricular drainage	5.6	ONSD > 5.6 mm disclosed a sensitivity of 93.75% and a specificity of 86.67% for identifying increased ICP (over 20 mmHg).
Liu D. et al., 2017 [30]	Prospective observational	110	Patients who underwent lumber puncture	5.6	A significant correlation was found between ICP and body mass index. A discriminant equation for predicting ICP = 0.169 × BMI + 1.484 × mean ONSD-12.74.
Ebraheim AM, et al., 2018 [31]	Prospective, observational	54	Idiopathic intracranial hypertension.	6.2	ONSD was significantly higher in patients compared to controls. ONSD could be a valuable noninvasive additional tool to diagnose and monitor IIH patients. IIH insignificantly influences ophthalmic vessels hemodynamics.
Kishk N. A. et al., 2018 [32]	Case-control	99	Idiopathic intracranial hypertension	6.05	The best estimated cut-off value of the ONSD in detecting IIH was 6.05 mm. The sensitivity and the specificity were 73.2% and 91.4%, respectively. ONSD but not OND/ONSD ratio could offer a bedside adjunct or alternative indicator of elevated ICP for these patients.
Onder H. et al., 2021 [33]	Prospective, observational	103	Idiopathic intracranial hypertension	6.3	The right ONSD values were higher in the IIH group. Using a cut-off of 6.3 mm, ONSD had the following performance characteristics: sensitivity 18%, specificity 81%.
Robba C et al., 2020 [8]	Prospective, observational	100	Neurological patients with invasive ICP monitoring for risk of	≥ 6.0	The area under the curve to estimate intracranial hypertension was 0.78 for ONSD. The multimodal combination of ONSD and eICP may increase the accuracy to estimate the occurrence of intracranial hypertension.
Robba C et al., 2017 [34]	Prospective, single-cohort observational	64	Brain injury requiring invasive ICP monitoring	5.85	ONSD is the best estimator of ICP. Model that best fitted the data: nICP ONSD : 5.00 × ONSD − 13.92mm Hg
Canakci Y. et al., 2018 [35]	Prospective, observational	100	patients who applied to the ER with the complaint of headache	≥5.0	In all cases with abnormal CT findings, ONSD measurements were significantly higher. ONSD value in the ipsilateral side with the lesion was significantly higher than the contralateral side.
Yazar M. À. et al., 2019 [36]	Prospective observational	45	Brain death	-	ONSD values of group brain (7.55 ±.29) death were significantly higher than both control group (5.07 ±.32) and comatose patients 6.99 ± .42.)
Robles-Caballero A. et al., 2021 [37]	Prospective cross-sectional	99	Brain death	-	ONSD values capable of recognizing CBF were not identified.
Ortner C. M. et al., 2018 [38]	Prospective observational cohort	95	Preeclampsia	5.8	ONSD were common in preeclampsia with severe features.
Simenc G. B. et al., 2018 [39]	Prospective, observational	60	Preeclampsia	5.8	ONDS diameter was significantly higher in patients with severe preeclampsia compared to controls before delivery, and one day and four days post-delivery
Sterrett M. E. et al., 2022 [40]	Cohort	62	Pregnant women	5.8	There was not a significant elevated optic nerve sheath diameter relative risk between severe preeclampsia patients with neurologic features and non-preeclampsia control patients
Ertl M. et al., 2018 [41]	Prospective, observational		Post cardiac arrest	5.75	Nonsurvivors showed significantly higher ONSD values (*P* < .001). For predicting mortality, a threshold of 5.75 mm was calculated with a specificity of 100%.
Yang 12/12/2022 11:50:00 AM Z et al., 2020 [42]	Prospective observational	90	Septic	5.5	The ONSD of patients with sepsis-associated encephalopathy was significantly wider than non-sepsis-associated encephalopathy patients.
Donovan J. et al., 2021 [43]	Prospective observational	107	Tuberculous meningitis	5.5	Higher baseline ONSD was associated with more severe disease and abnormal brain imaging and increased death by 3 months.

The search strategy was the MESH terms of “intracranial hypertension AND Optic nerve sheath diameter”. In total, 512 studies were found in the last 5 years. Studies were excluded if duplicated (1), in non-English language studies (5), trial/protocol registration (4), case report (12), studies in healthy subjects (27), non-ultrasound (54), reviews (39), comments/responses (60), ophthalmologic pathology (38), including children (30), animals (147), concordance between other image methods (49), *N* < 45 patients (20), and retrospective (2). Hence, 24 publications were eligible for summarization in this table

*GCS*, Glasgow Coma Scale; *ONDS*, optic nerve sheath diameter; *ODE*, optic disc elevation; *OND*, optic nerve diameter; *ER*, emergency room; *ICP*, intracranial pressure; *IIH*, idiopathic intracranial hypertension; *eICP*, estimate intracranial pressure; *ROC*, receiver operating characteristic curve; *CBF*, cerebral blood flow velocity

#### Technique

**Fig. 4:**
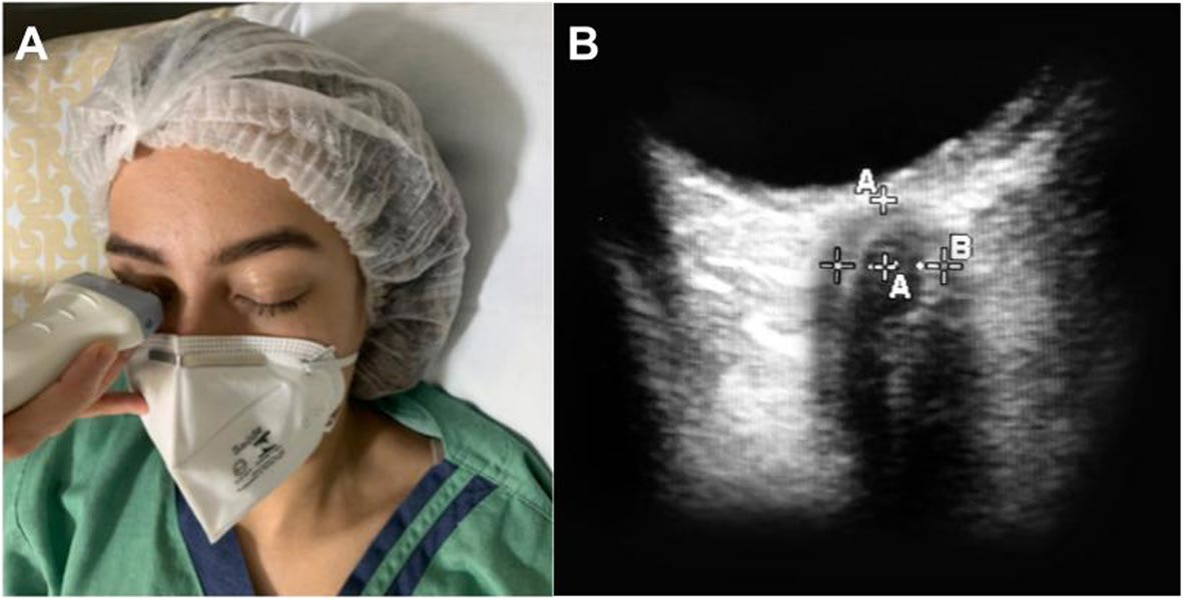
**A** The Nerve optic measured through the transorbital window. **B** ONSD is measured 3mm behind the globe, using an electronic caliper and an axis perpendicular to the optic nerve

The ultrasound probe (linear probe) should be placed on the temporal area of the eyelid (Fig. 4). The probe should be then angled in order to display the optic disc and the entry of the optic nerve into the globe. ONSD is measured 3mm behind the retina, using an electronic caliper and an axis perpendicular to the optic nerve (Fig. 4). It is paramount to perform a short examination duration and minimize image information loss by recording an US video clip to reduce the examination duration for the safety of the patient, ALARA principle (as low as reasonable achievable) [44]. It is important to reduce the power output of the US, usually expressed as a percentage.

To reduce errors, it has been suggested to use the mean of three values obtained from each patient, for each eye. Furthermore, identifying the main structures (optic nerve, retina, meninges) and following the CLOSED protocol help to improve the inter- and intra-rater reliability and decrease the risk of inter-rater bias [44, 45]. At present, ONDS is considered a basic-plus skill [11].

With regard to ONSD measurement, in the vast majority of published studies, ONSD is defined as the distance between the external borders of the hyperechogenic area surrounding the optic nerve, which represents the subarachnoidal space including the arachnoid mater. In some studies, however, the ONSD was also measured including the outer hypoechogenic rim, which represents the dura mater, resulting in a large heterogeneity of reported reference values [20, 22].

The cutoff of ONDS is another lack in the literature. An ONSD >5.8 mm indicates increased ICP with a sensitivity of 90% and a specificity of 84%; however, the cut-off value is divergent in literature (Table 2), and recent guidelines suggest a threshold ≥ 6.0 mm [5].

## Facilities using brain ultrasound appraisal

### Noninvasive intracranial pressure

**Fig. 5:**
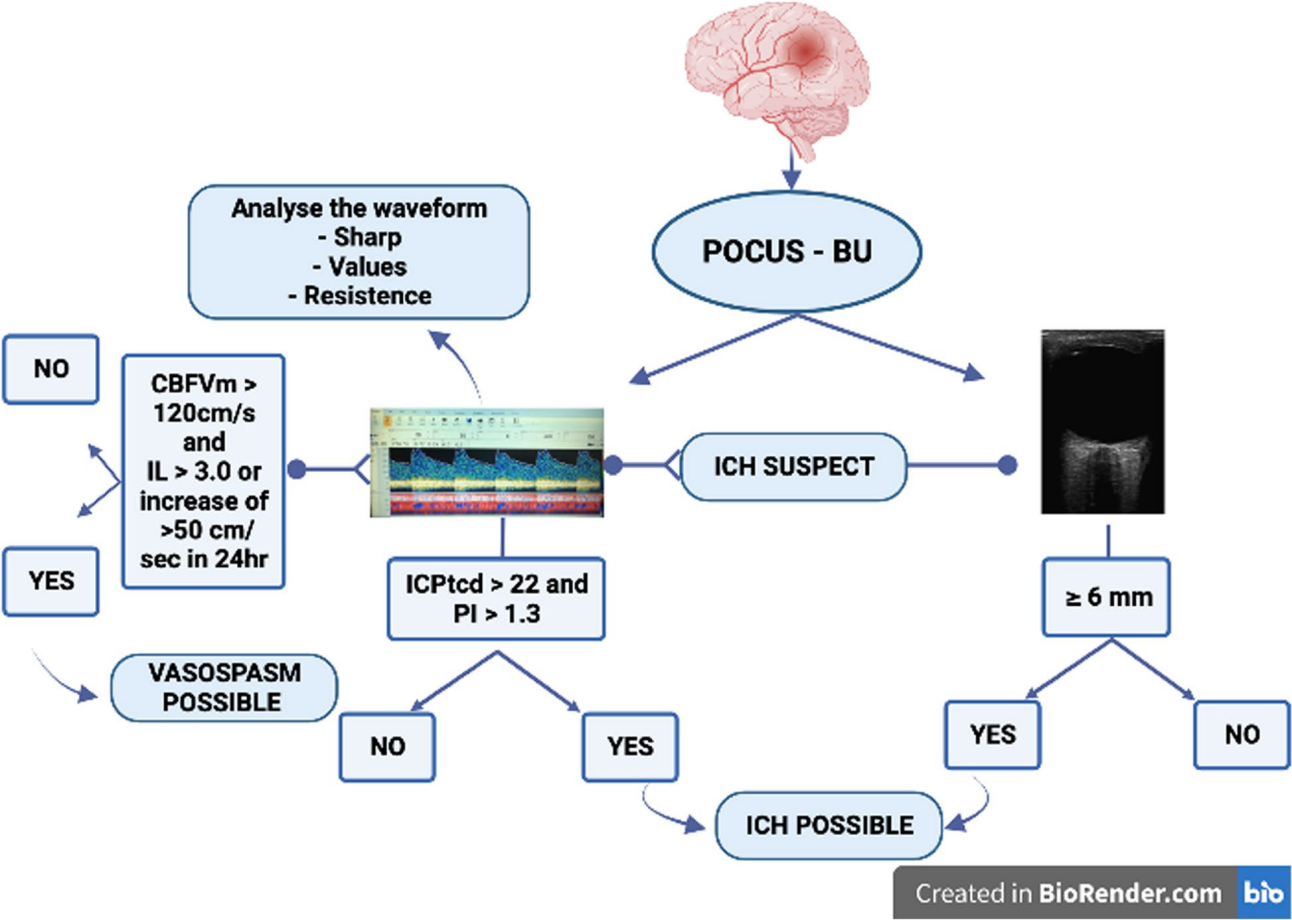
An algorithm for evaluating intracranial pressure (ICP) and vasospasm by brain ultrasound. CBFV, cerebral blood flow velocity; ICPtcd, intracranial pressure through transcranial Doppler; PI, pulsatility index; ICH, intracranial hypertension. LI, Lindegaard index = CBFVm of middle cerebral artery (MCA)/CBFVm of the extracranial internal carotid artery

The first practical use of BU at bedside is the access to indirect and non-invasive information of the intracranial pressure through ONSD and TCD measurements. The association of information such as ONSD ≥ 6mm, PI > 1.4, and ICPtcd >22 reinforce intracranial hypertension suspicion (Fig. 5). The waveform analysis of the MCA (a sharp decrease of diastolic flow velocity, P2>P1) is also another tool to raise suspicion of increased ICP (Fig. 5). This non-invasive tool is paramount information about cerebral compliance, especially when invasive access to intracranial pressure is not available.

### Hydrocephalus

**Fig. 6:**
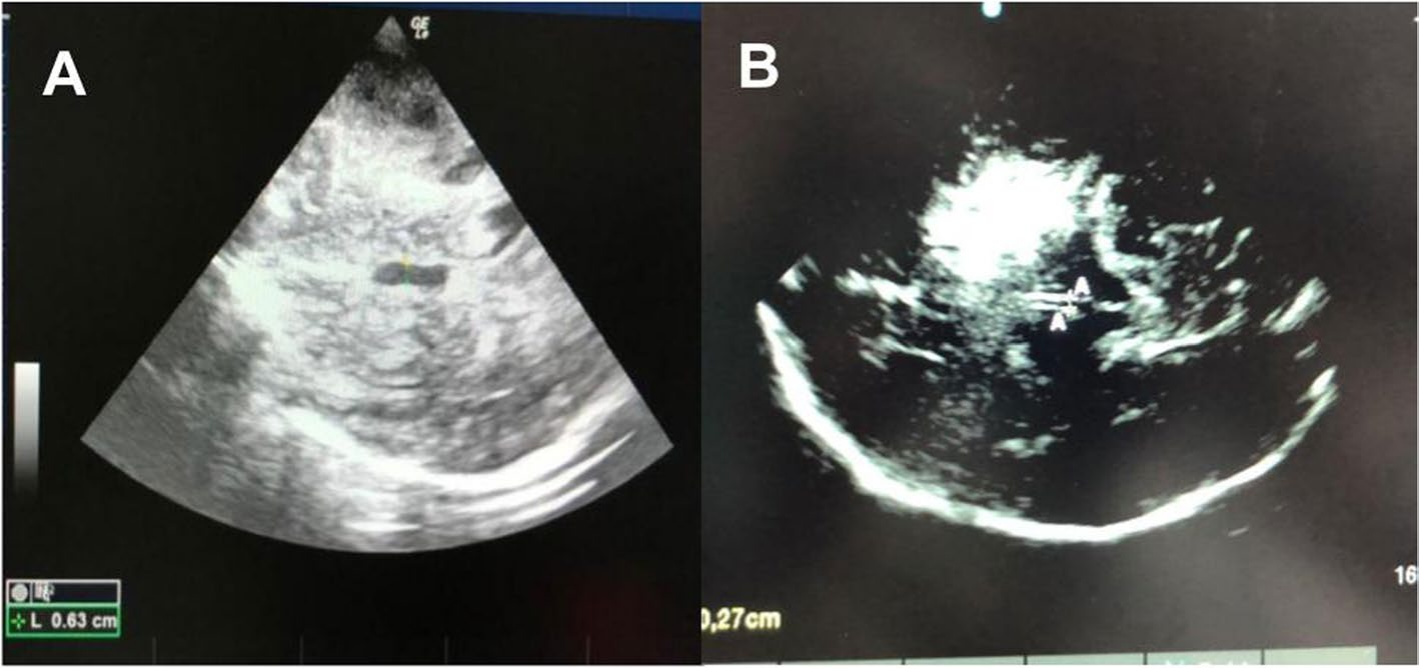
Diencephalic plane, the third ventricle is obtained (A-A). It should be measured the largest transverse diameter of the third ventricle with its hyperechogenic margins. These figures were performed of two women patients, with same ages, 24h after derivation devices were removed. **A** Large third ventricles suggesting hydrocephalus. **B** Normal value without hydrocephalus

Several neurocritical patients have a high risk to develop hydrocephalus. BU through the estimation of the size of the third and lateral ventricles can provide important information about hydrocephalus (Figs. 3 (B) and 6). Intracranial ventricles appear as double hyperechogenic lines, thus making feasible the evaluation of their diameter. It has been observed a good correlation between BU and CT measurements of the width of the third ventricle (*r* = 0.83–0.95), of the right (*r*=0.86) and left (*r*=0.92) frontal horns, and middle part (*r*=0.73) of the lateral ventricles [46].

It is important to note that this technique is useful to evaluate a trend of ventricles size especially after derivation devices removal, in order to minimize transfer of the patients and ICU staff team to CT exams. Although the assessment of third ventriculus is considered a basic plus skills, the evaluation of hydrocephalus needs more experience and it is considered as pre advanced skills [11].

### Guided ventricular puncture

Although insertion of an external ventricular derivation (EVD) is generally safe, complications such as hemorrhage and suboptimal placement have been reported in up to 40% of cases [47]. This led several authors to suggest that current ventriculostomy practice leaves room for improvement and calls for wider adoption of image-guided insertion, especially in patients with smaller ventricles and midline shift. In this setting, the BU could provide real-time information and can optimize the “standard” ventriculostomy trajectory [48].

After bone removal, the transducer should be placed over the dura mater, identifying the mesencephalic plane and its landmark of the midbrain (“butterfly” or “mickey”) (Fig. 3 (C)). An approximate 10–20° cranial tilting of ultrasound beam allows identification of the third and the ipsilateral frontal horn of the ventricle, before the EVD catheter placement. Dimming of the overhead room lights helps facilitate improved visualization of the ultrasound images (Additional file 1: Video 1 and Additional file 2: Video 2).

### Brain midline shift

Brain midline shift (MLS) through BU has been described since 1996 by Seidel et al. [49]. Using 2–2.5-MHz sectorial transducer probe, the distance between the BU and the center of the third ventricle is measured through a line perpendicular to the walls of the third ventricle from both the ipsilateral and contralateral sides, and the deviation from the presumed midline is calculated by the equation MLS=(A−B)/2 [46].

The correlation of MLS (> 2.5 mm) apparent on CT and BU in acute severe MCA territory stroke is high and presents sensitivity of 83% and 100% of specificity [50, 51]. Another study showed that an MLS >0.35 cm on BUS in severely ill neurosurgical ICU patients, including patients after decompressive craniectomy or with subcutaneous temporal hematomas, can predict a MLS >0.5 on CT with good sensitivity, specificity, and a positive likelihood ratio of more than 5 [9].

Bedside evaluation of MLS can be worthwhile to detect early cerebral complications and the need for additional imaging test or neurosurgical intervention for patients with significant intracranial mass effects. This skill is considered a basic plus [11].

### Assessment of cerebral autoregulation

Cerebral autoregulation (CA) refers to the ability of the brain to maintain approximately constant blood flow in response to changes in blood pressure [52].

Assessing CA remains a major challenge, with multiple methods and approaches having been described in literature [53]. Two distinct ways to assess CA have been studied: static and dynamic. Static cerebral autoregulation (sCA) [54] refers to changes in blood flow in response to changes in blood pressure in the “steady state” (i.e., where mean blood pressure changes to a new level and is held there for minutes or hours). In dynamic cerebral autoregulation (dCA), the transient responses of blood flow to spontaneous changes in blood pressure are studied using a software which correlates changes in FV and blood pressure providing a correlation index [54].

BU allows the evaluation of CA in both static and dynamic ways (Table 3). Analysis of CA is considered a pre-advanced skill [11]; however, the noninvasive and bedside characteristics of BU are essential for critical care patients, which entails a growing interest in this field, both in neurological [60–63] and general critical care patients [53, 64, 65].

**Table 3:** Indices of static and dynamic CA used in the literature

Index	Definition	Static/dynamic	Impaired CA	BU
THRT [55]	Changes in cerebral blood flow velocity after a brief compression of the ipsilateral common carotid artery. THRT = FVsystolic (hyperemia)/FVsystolic (baseline)	Static	≤ 1.1	Yes
ARI [54]	Autoregulation index derived from cerebral blood flow velocity and transfer function analysis	Dynamic	≤ 4	Yes
PRx [56]	Moving Pearson’s correlation coefficient between intracranial pressure and blood pressure.	Quasi- Static	≥ 0.3	No
Mx/Mxa [57]	Moving Pearson’s correlation coefficient between cerebral blood flow velocity and blood pressure	Quasi- Static	≥0.3	Yes
COx [58]	Correlation coefficient between MAP and rScO2	Static	+1	No
RoR [59]	Ratio of slope of cerebral blood flow velocity recovery normalized by blood pressure after thigh cuff release	Dynamic	-	Yes

*BU*, brain ultrasound; *THRT*, transient hyperemic response test; *FV*, flow velocity; *ARI*, autoregulation index; *PRx*, pressure-reactivity index; *Mx*, Mean index; *COx*, cerebral oximetry index; *rScO2*, regional cerebral oxygen saturation; *RoR*, rate of regulation

In addition to the analysis of whether or not there is impairment in CA, its monitoring can provide an individual “optimal” cerebral perfusion pressure (CPPopt) target at which CA is best preserved [66].

## Clinical applications

### Traumatic brain injury

Early investigation of traumatic brain injury (TBI) patients by using BU may provide important information regarding CBF and ICP [67]. Martin et al. have described some BU patterns of cerebral blood flow occurring in 3 phases after TBI: first 24 h of hypoflow, then the following 3 days of hyperemia and the last 10 days of vasospasm [68]. Sonographic vasospasm can be present in about 40% of severe TBI, even without traumatic subarachnoid hemorrhage (SAH) [69]. The presence of hypoflow, hyperemia, or vasospasm, independently of the period, is associated with unfavorable outcomes [67]. Ract et al. have described that half of severe TBI patients will present some pathological findings in BU [70]. Moreover, a mean CBFV < 30 cm/s or diastolic flow velocity < 20 cm/s or pulsatility index > 1.4 in the MCA may indicate compromised cerebral hemodynamic and potentially increased ICP [71].

When analyzing CBFV by BU/TCD, we must consider occasional oscillation in PaCO_2_, not only in TBI but in all brain injury patients. Hypocapnia stimulates cerebral vasoconstriction that can reduce CBFV, and the inverse occurs in the opposite situation [71].

### Subarachnoid hemorrhage (SAH)

SAH is a complex disease, and depending on the phase of SAH, BU can detect failure of CA, sonographic vasospasm, and signals of intracranial hypertension due to hydrocephalus or delayed cerebral ischemia (DCI) [41, 42]. BU is a tool which adds important information to the constellation of important clinical information, aimed at the early detection, intervention, and guiding intervention designed to prevent the neurologic sequelae related to cerebral vasospasm and delayed cerebral ischemia, especially in poor-grade patients, whose clinical examination is jeopardized.

A recent meta-analysis regarding the topic of vasospasm and SAH confirmed that MCA is the artery with the highest prevalence of vasospasm, comprising around 70% of all cases. Therefore, POCUS-BU, through the insonation of MCA [6], may be used to identify patients with vasospasm with a high positive predictive value, but with low negative predictive value and sensitivity. The majority of studies included in this meta-analysis reported CBFV of 120 m/s in MCA, with 66.7% of sensibility and 89.5% specificity [6]. It is important to differentiate hyperemia from vasospasm, and therefore the Lindegaard [17] and Soustiel [18] indexes should be measured. Nevertheless, a rapid increase of 50cm/sec or more over a 24-h period seems to be a strong predictor of neurologic deficit of DCI (Fig. 5) [72].

### Brain death diagnosis

The use of ancillary testing for brain death confirmation remains controversial, and some countries such as the UK and the USA do not require these tests to confirm brain death [73]. Brain death is a clinical diagnosis, and ancillary tests should always be considered supplementary and never a substitute. However, ancillary tests remain essential in brain death confirmation in peculiar cases, when clinical assessment is not reliable, such as clinical instability during apnea test, barbiturate therapy, hypothermia precluding proper brain death confirmation, extensive faciomaxillary injuries, and some cases of pediatric hypoxic brain injury [74]. On the other hand, in countries such as Brazil and Australia, an ancillary test is always required to confirm brain death [75]. In this context, BU/TCD is a useful alternative, due to being safe, noninvasive, and conducted at the bedside.

TCD can confirm brain death by evaluating cerebral circulatory arrest, which has distinctive flow patterns: oscillatory flow representing reversal of diastolic flow and systolic spikes representing lack of net forward flow. In a recent meta-analysis [74], TCD as ancillary testing for brain death was found to be highly sensitive (89%) and specific (98%). Nevertheless, TCD is an operator-dependent, experience-requiring technique, and false-negative cases may occur in patients with poor transcranial windows for ultrasound insonation, which occurs in 10–20% of cases due to the thickness and porosity of the skull bone [76]. In addition, some patients with clinical diagnosis of brain death may present residual blood flow even after cerebral arteriography, such as decompressive surgery, ventricular shunt, and in anoxic encephalopathy after cardiac arrest.

**Fig. 7:**
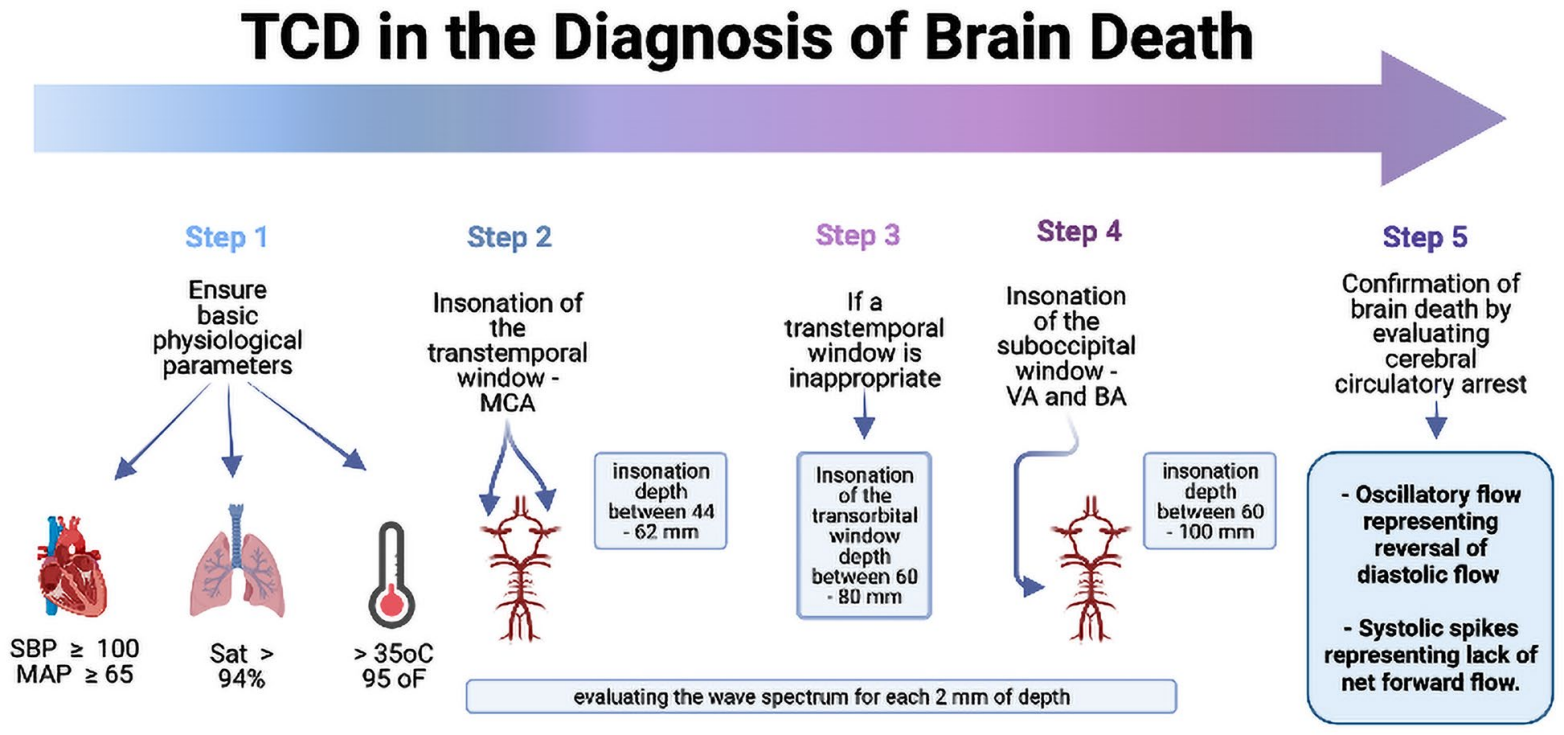
A step-by-step algorithm for TCD use in the diagnosis of brain death. SBP, systolic blood pressure; MAP mean arterial pressure; Sat, saturation of oxygen; MCA, middle cerebral artery; VA, vertebral artery; BA, basilar artery

The same physiological prerequisites before beginning the protocol for brain death must be maintained, such as systolic blood pressure above 100 mmHg, avoidance of hypoxemia (SpO2>94%), and no hypothermia (>35.1) [77]. In addition, the patient must be in the dorsal decubitus position. The following steps should be followed for applying TCD as a diagnostic test to confirm brain death in Brazil (Fig. 7) [75].

TCD is one of the most appropriate ancillary tests when clinical conditions or medications make the clinical examination unsuitable, or in countries in which it is mandatory.

### Monitoring brain function in general critical care unit

Brain dysfunction is observed as part of multiorgan dysfunction syndrome in critical care patients in a number of pathologies such as sepsis, shock, and respiratory failure [78, 79].

The traditional focus in critical care is on monitoring hemodynamic and respiratory systems; nevertheless, neuromonitoring should also become part of the standard care to maintain brain homeostasis in the critical care setting.

### Acute respiratory distress syndrome (ARDS)

**Fig. 8:**
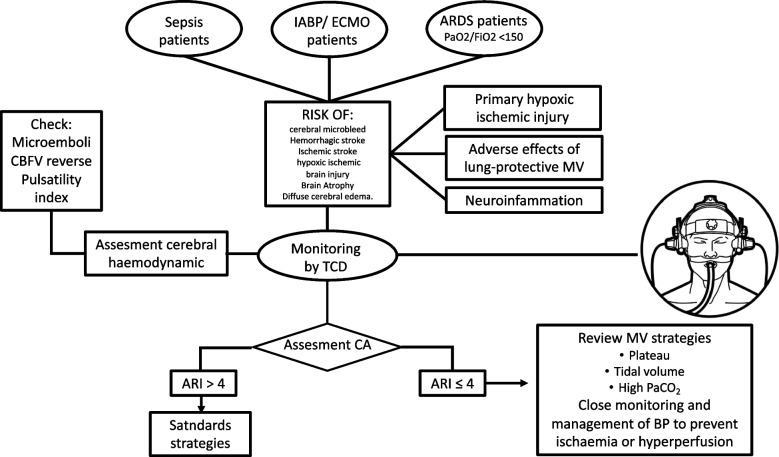
Monitoring of cerebral blood flow regulation in critical care scenarios. ARDS, acute respiratory distress syndrome; IABP, intra-aortic balloon pump; ECMO, extracorporeal membrane oxygenation; TCD, transcranial Doppler; CBFV, cerebral blood flow velocity; ARI, autoregulation index; CA, cerebral autoregulation; MV, mechanical ventilator; PaCO2, partial pressure of carbon dioxide

ARDS has been associated with a high incidence of neurological complications and secondary acute brain injury [80]. The physiopathology is not well understood; however, the primary hypoxic-ischemic injury from hypoxic respiratory failure and secondary injury from lung injury induces neuroinflammation and increased intracranial pressure from lung-protective mechanical ventilatory strategies. Monitoring using BU is possible in real time, to identify patients who are at risk of presenting cerebral blood flow disturbances. Although the main goal of the management of these patients is the lung, in high-risk patients for brain damage, an approach which takes into consideration also the cerebral dynamics and needs is warranted (Fig. 8).

### Sepsis

Sepsis and shock patients are also other examples of critical care patients where neurological complications are common [81]. Impairment of CA has also been showed in these patients and it was demonstrated to be associated with systemic organ [82] and brain dysfunction [83]. Furthermore, a systematic review and meta-analysis reported that abnormal PI values are often observed in sepsis patients. In early sepsis, the PI seems to increase, and on the other hand, PI reduction was found to be very common in the later phases of sepsis [84]. BU can be used as part of multimodal neuromonitoring to assess cerebrovascular resistance, CA, as well as the optimal CPP to apply to this group of patients.

### Dispositive devices for hemodynamic support

The intra-aortic balloon pump (IABP) and extracorporeal membrane oxygenation (ECMO) are often used to manage patients with advanced heart failure, cardiogenic shock, and ARDS patients [85]. Despite both devices are linked with neurological complications which significantly affect the short-and long-term outcomes of patients, there is sparse data on systematic neurological monitoring in this population [85].

Diastolic flow reversal in CBFV has been described in patients with IABP [86]. This pattern of flow has also been associated with intracranial hypertension, brain death, and comatose patients, and it is probably due to rapid deflation of IABP [86], with the suggestion that CBFV reversal is iatrogenic and should be avoided. One possible approach is to optimize the balloon inflation/deflation cycle, by moving deflation to the absolute end of diastole [86]. Nevertheless, the largest study published with TCD in IABP patients did not show deterioration of CA [87].

BU monitoring is also important to detect microemboli in IABP and ECMO, a cause of cerebral infarct, thromboembolism, and commonly iatrogenic in origin [88]. With the increasing application of ECMO, more attention has been paid to the neuromonitoring devices. In a prospective observational study in patients with ECMO, 18 thromboembolic events were observed, 14 of which had positive microembolic signals in TCD [89], and in a recent systematic review, TCD by microemboli and CA monitoring appeared to have potential for assessing the risk of ischemic stroke [88].

It is important to note that new evidence suggested that CA is often impaired in critical care patients [64, 65, 82, 83], and it could play a role in the occurrence of brain dysfunction leading to brain ischemia or hyper-perfusion causing edema and capillary damage [90]. In these critical care patients, a closer analysis of hemodynamic changes, CO2, temperature, drugs, and other physiological variables is deemed even more important.

Although the CA analysis still awaits prospective clinical trials to evaluate outcomes and establish the prognostic value of CA [4], BU may allow for adequate therapeutic measures to be taken through non-invasive assessment of cerebral circulatory status in critical care patients in real time and being as a continuous methods (Fig. 8).

Some studies have been published regarding ABPopt in general critically ill patients using BU as a tool [66, 91]. The idea of treating patients with an optimal cerebral autoregulation “or optimal BP” regime is not firmly established. Nevertheless, it seems feasible and large deviation from CPPopt has been associated with adverse outcomes [66].

## Conclusions

Considering the ample use of ultrasound in critical care scenarios and the benefits it entails, such as bedside performance, repeatable after therapies, and easy follow-up as a noninvasive method and without requiring contrast, it is unquestionably the best method to provide intracranial hemodynamics information in real time. Although expertise requires the apprehension of specific skills acquired through knowledge of the various landmarks, parameters, and fields of application, and especially through hands-on training and practice of this technique, the POCUS-BU deserves to be more explored in a critical care setting and more studies are deemed necessary to this end.

## Supplementary Information

**Additional file 1: Video 1**.

**Additional file 2: Video 2**.

## Data Availability

Not applicable.
